# Supplementary material for: Structure of a transcribing Pol II-DSIF-SPT6-U1 snRNP complex
Source: Nat Commun. 2025 Jul 1;16:5823. doi: 10.1038/s41467-025-60979-9 (PMC12216098; doi:10.1038/s41467-025-60979-9)
Supplement: Supplementary file 1 — Supplementary Information [file 41467_2025_60979_MOESM1_ESM.pdf]

Supplementary Information

Structure of a transcribing Pol II-DSIF-SPT6-U1 snRNP complex

Luojia Zhang[1], Christopher Batters[1], Shintaro Aibara[2], Yuliya Gordiyenko[1], Kristina Žumer[2], Jana Schmitzová[2], Kerstin Maier[2], Patrick Cramer[2], Suyang Zhang[1*]

[1]MRC Laboratory of Molecular Biology, Cambridge, CB2 0QH, United Kingdom

[2]Max Planck Institute for Multidisciplinary Sciences, Göttingen, 37077, Germany

*Corresponding author. Email: szhang@mrc-lmb.cam.ac.uk

The PDF file includes:

Supplementary Figs 1 to 6

Supplementary References

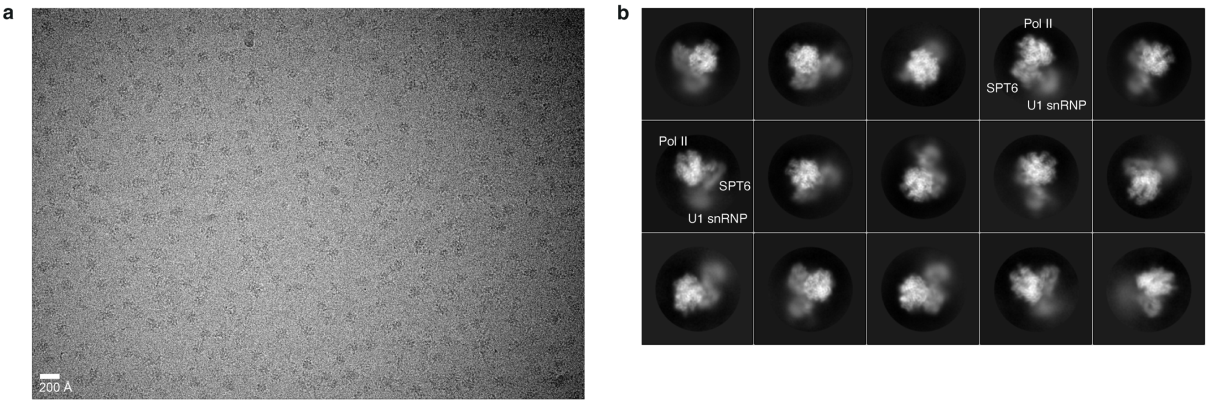

Supplementary Fig. 1 | Cryo-EM analysis of the EC-DSIF-SPT6-U1 snRNP complex. a, Representative micrograph of the EC-DSIF-SPT6-U1 snRNP complex collected on the 300 kV Titan Krios with the K3 detector in electron counting mode. A total of 18,830 image stacks were collected. **b**, Two-dimensional averages of the EC-DSIF-SPT6-U1 snRNP complex.

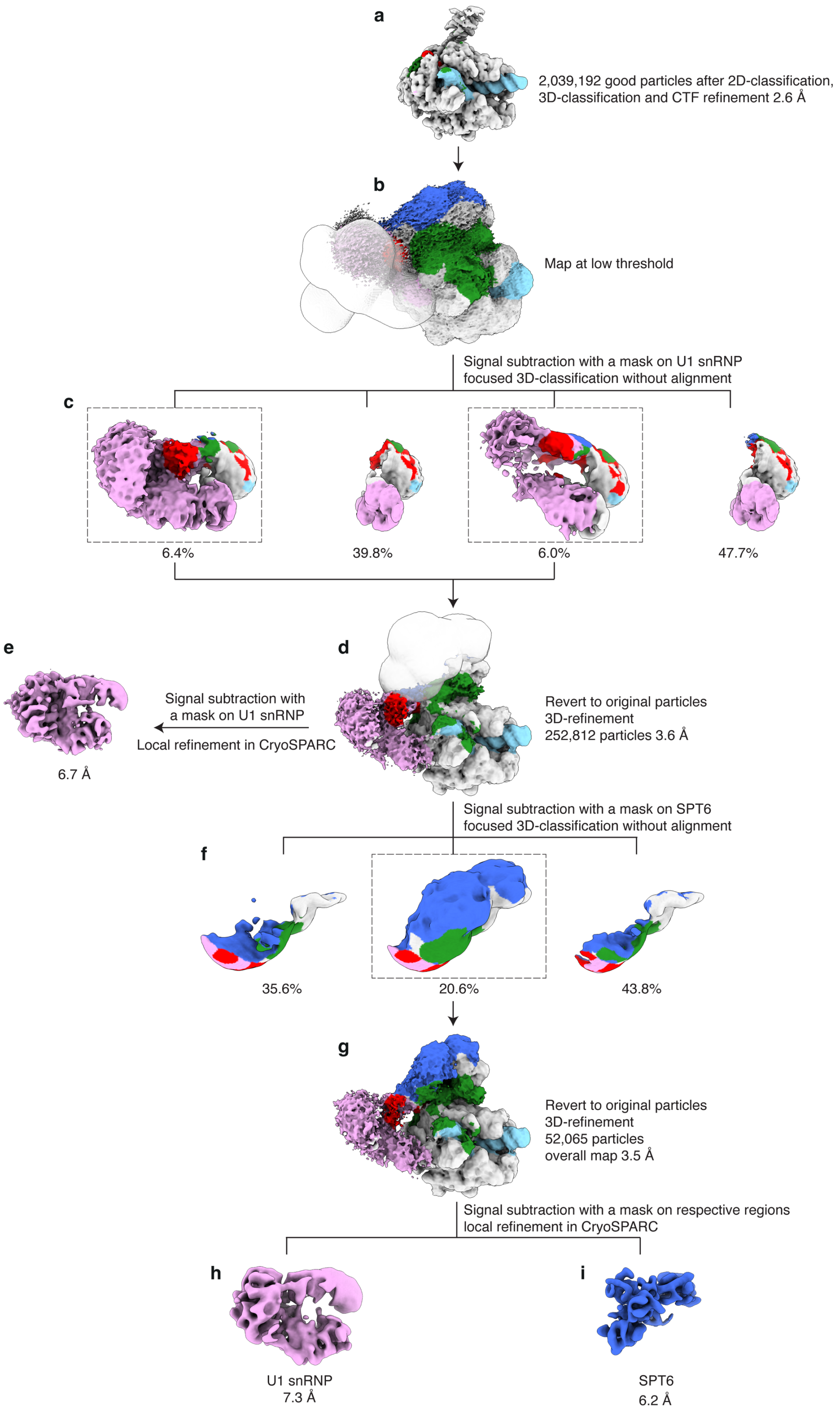

Supplementary Fig. 2 | Cryo-EM data processing of the EC-DSIF-SPT6-U1 snRNP complex. 2D-classification, followed by 3D-refinement and 3D-classification with fine-angle sampling was performed to remove bad particles from the dataset (**a**). Signal subtraction

followed by focused 3D-classification without alignment was performed using a soft mask on U1 snRNP (**b, c**). Particles containing densities of U1 snRNP were reverted to original particles and 3D-refined, resulting in a reconstruction of 3.6 Å resolution (**d**), with a locally refined map at 6.7 Å for U1 snRNP (**e**). Following signal subtraction and focused 3D-classification without alignment with a soft mask on SPT6 (**f**), particles containing densities of SPT6 were reverted to original particles and 3D-refined. This results in a final reconstruction of EC-DSIF-SPT6-U1 snRNP at an overall resolution of 3.5 Å (**g**). Particle subtraction followed by local refinement improved the local resolution of U1 snRNP (**h**) and SPT6 (**i**). The cryo-EM densities were colored according to Fig. 2c.

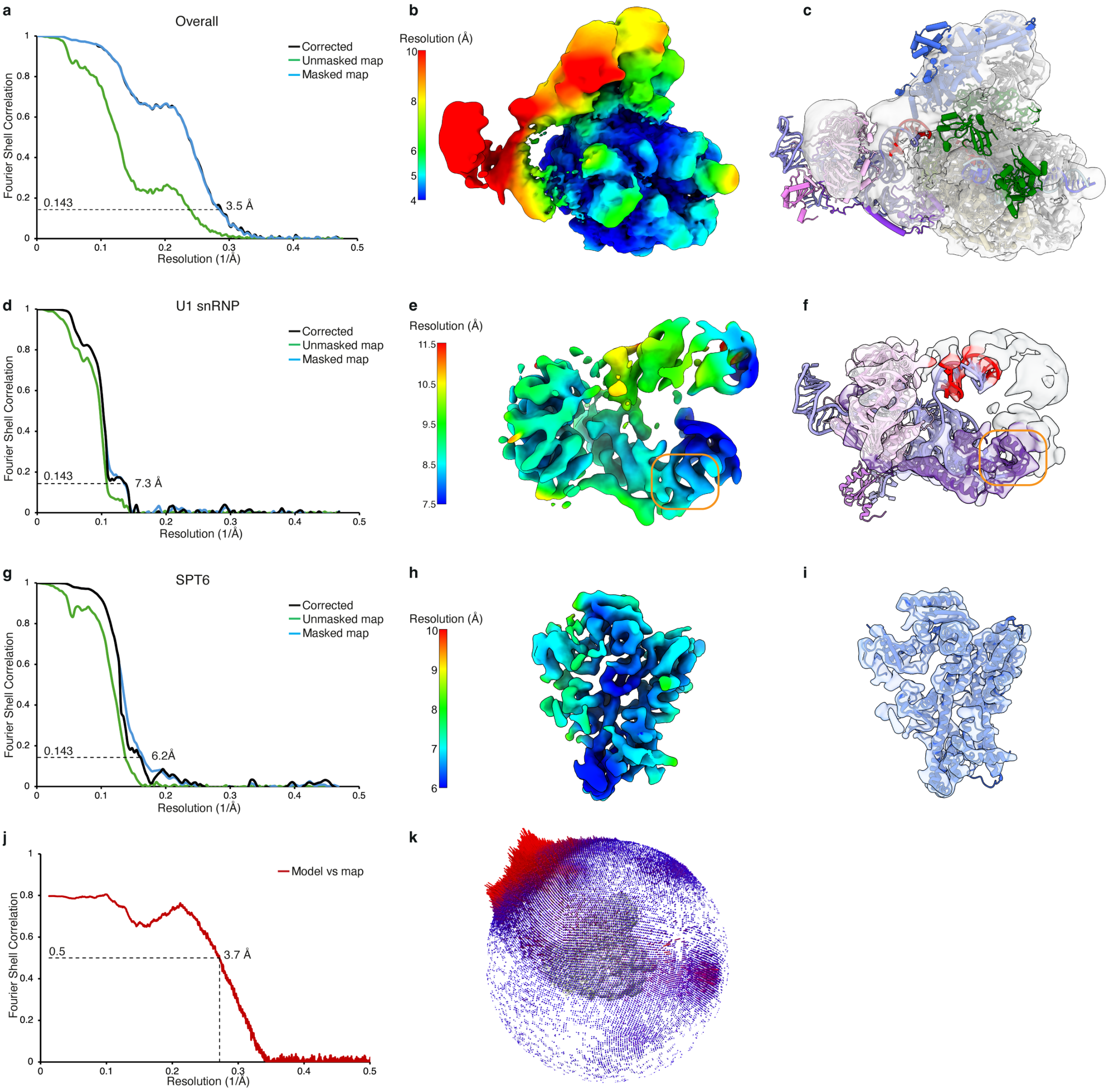

Supplementary Fig. 3 | Cryo-EM densities, local resolution estimation and FSC curves of the EC-DSIF-SPT6-U1 snRNP complex. a-i, Gold-standard Fourier Shell Correlation (FSC) curves, local resolution estimations and cryo-EM densities with fitted models for the overall map (**a-c**), U1 snRNP (**d-f**) and SPT6 (**g-i**). The orange boxes in **e** and **f** indicate the Pol II-U1 snRNP interface. The locally filtered and sharpened map was shown for the overall map, and sharpened maps were shown for the focused refined maps of U1 snRNP and SPT6. **j**, Model versus map FSC for the locally filtered and sharpened overall map using the FSC standard of 0.5. **k**, Angular distribution plot of the overall map (grey).

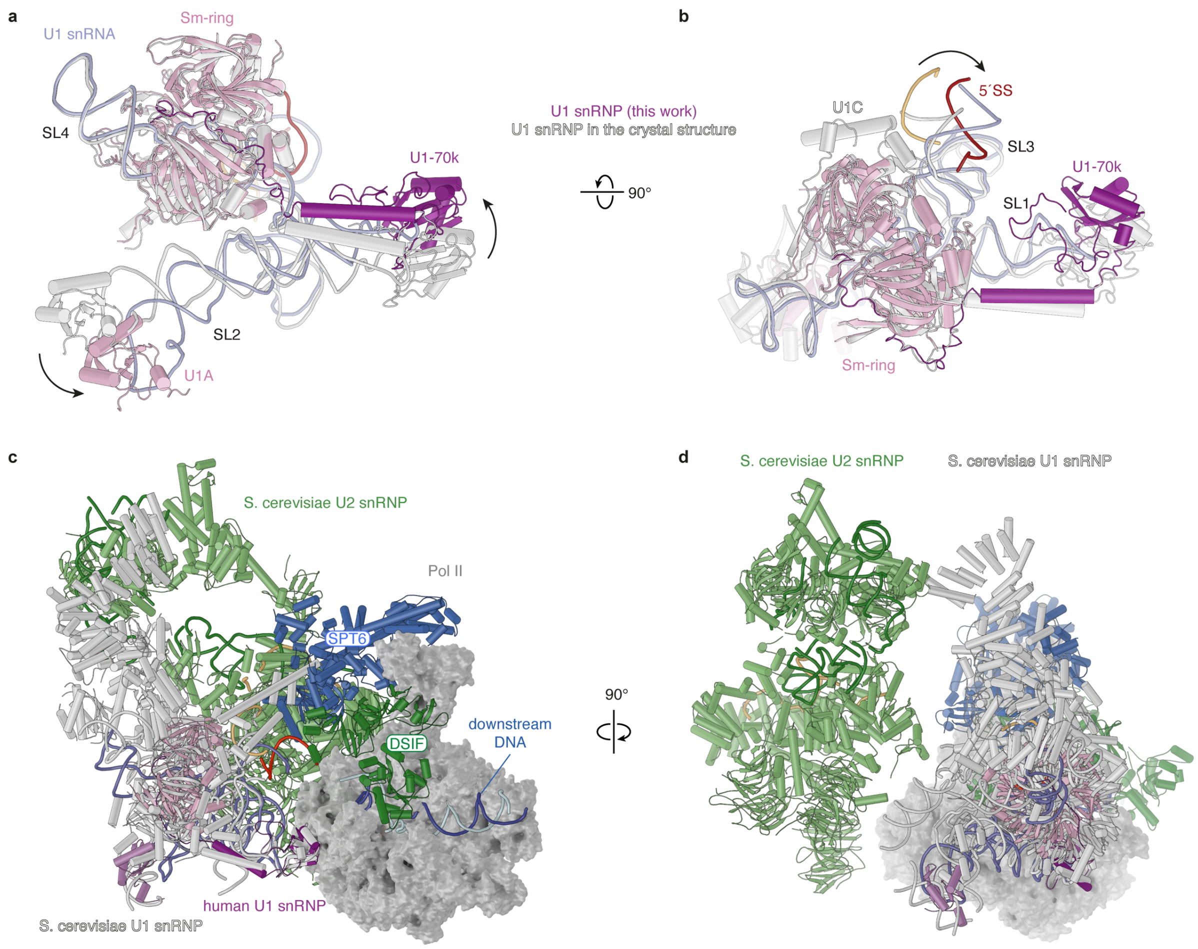

Supplementary Fig. 4 | Superposition with previous spliceosome structures. a, U1 snRNP model of the EC-DSIF-SPT6-U1 snRNP (colored as Fig. 2c) is superimposed onto the U1 snRNP crystal structure (PDB: 3CW1 in white)[1] on the Sm-ring, showing movements of stem loops with associated proteins. **b**, The 5´SS-U1 snRNA duplex (red and slate) rotates away from the Sm-ring, leaving a bigger gap at the binding site for U1C. 5´SS of the crystal structure is colored orange. **c**, **d**, Superposition of the EC-DSIF-SPT6-U1 snRNP structure with the yeast pre-spliceosome (PDB: 6G90)[2] on U1-70K indicates that pre-spliceosome formation may occur on the Pol II surface (grey surface). Yeast U1 snRNP is shown in white and U2 snRNP in light green. The pre-mRNA of the EC-DSIF-SPT6-U1 snRNP complex is depicted in red, while that of the yeast pre-spliceosome in orange. A minor clash with SPT6 was observed for an unassigned helix of yeast U1 snRNP which is not an integral part of human U1 snRNP.

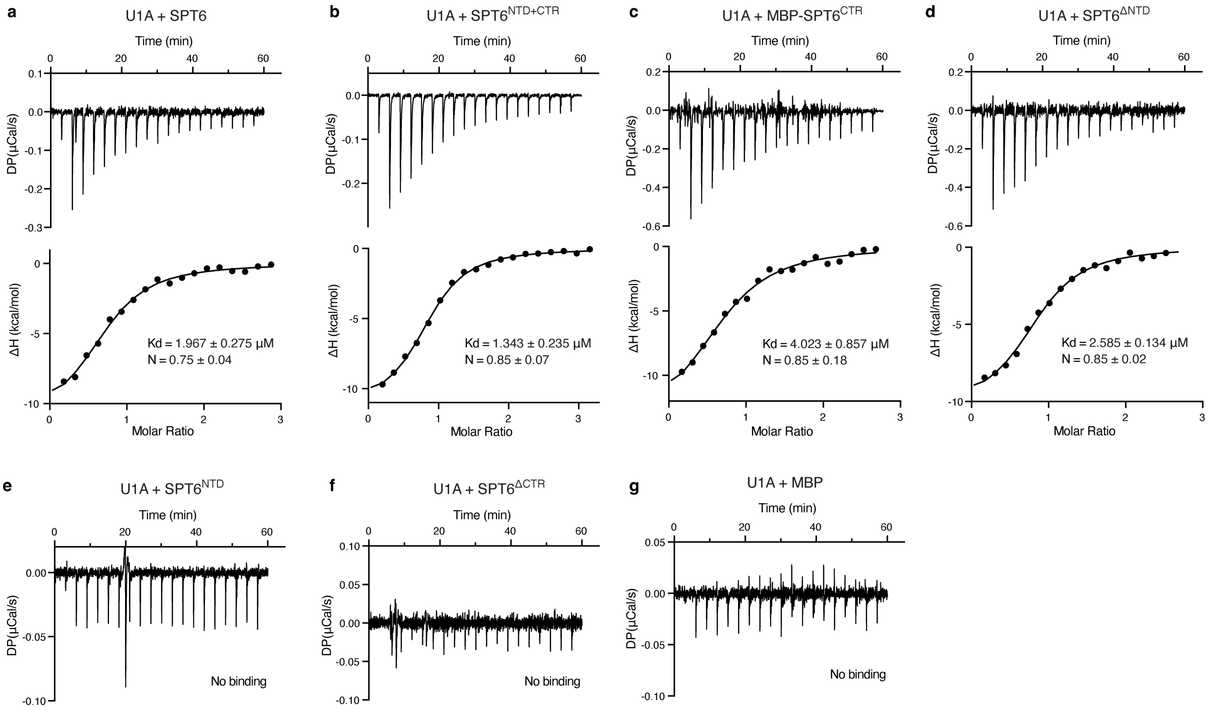

h

Protein 1	Protein 2	Kd (μM)	N (sites)
U1A	MBP	No binding	
U1A	SPT6	1.967 ± 0.275	0.75 ± 0.04
U1A	$SPT6^{NTD+CTR}$	1.343 ± 0.235	0.85 ± 0.07
U1A	$MBP\text{-}SPT6^{CTR}$	4.023 ± 0.857	0.85 ± 0.18
U1A	$SPT6^{\Delta CTR}$	No binding	
U1A	$SPT6^{NTD}$	No binding	
U1A	$SPT6^{\Delta NTD}$	2.585 ± 0.134	0.85 ± 0.02

Supplementary Fig. 5 | ITC analysis for the interaction between U1A and SPT6. a-g, Isothermal titration calorimetry (ITC) thermograms of U1A binding to SPT6 (**a**), $SPT6^{NTD+CTR}$ (**b**), His_6-MBP-tagged $SPT6^{CTR}$ (**c**), $SPT6^{\Delta NTD}$ (**d**), $SPT6^{NTD}$ (**e**), $SPT6^{\Delta CTR}$ (**f**) and MBP as a negative control (**g**). The upper panels show representative thermograms. The lower panels show the integrated heat changes and fitting of the data. **h**, Summary of the dissociation constant (Kd) and stoichiometry (N) for U1A-SPT6 interactions. Three independent biological replicates were performed, and data are presented as mean values ± standard deviation. Source data are provided as a Source Data file.

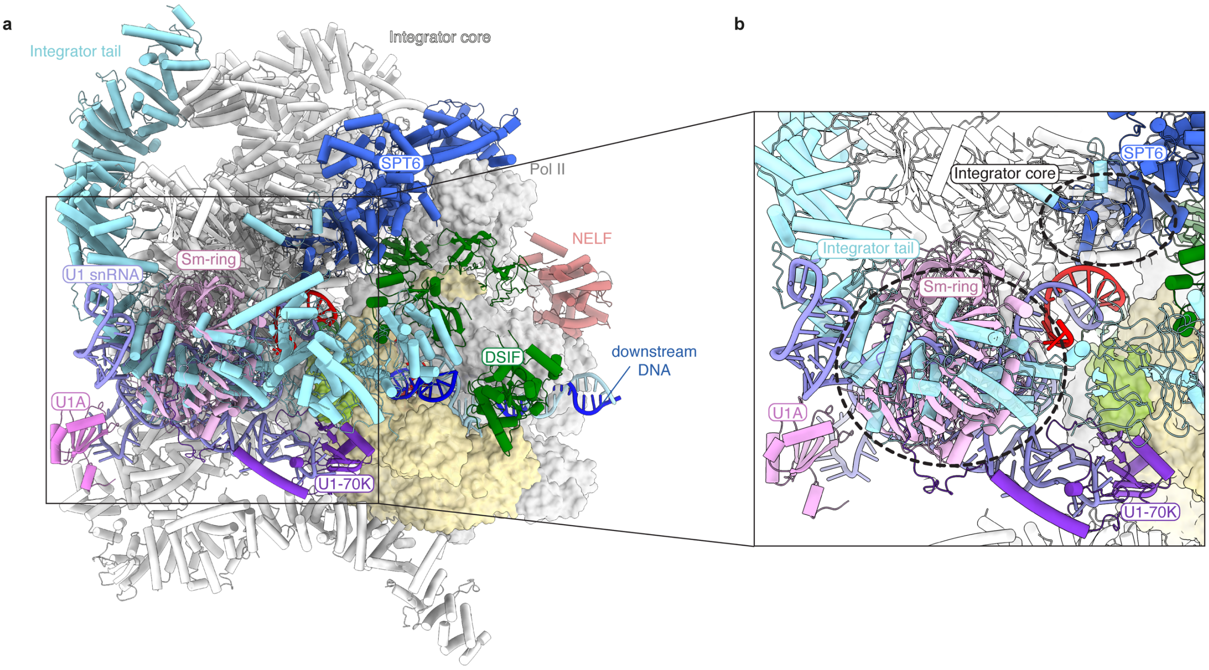

Supplementary Fig. 6 | The integrator tail module clashes with U1 snRNP on Pol II. a, Superposition of the integrator-containing pre-termination complex (PDB: 8RBX)[3] with the EC-DSIF-SPT6-U1 snRNP structure reveals that the integrator tail module (INTS10-13-14-15, in cyan) clashes with U1 snRNP on Pol II. **b**, Close-up view shows the clashes between the integrator tail module (cyan) and the Sm-ring (pink) and U1 snRNA (slate) and the clashes between SPT6 core (blue) and the integrator core module (white). Clashing regions are highlighted with dashed black circles.

Supplementary References

1. Pomeranz Krummel DA, Oubridge C, Leung AK, Li J, Nagai K. Crystal structure of human spliceosomal U1 snRNP at 5.5 A resolution. *Nature* **458**, 475-480 (2009).

2. Plaschka C, Lin PC, Charenton C, Nagai K. Prespliceosome structure provides insights into spliceosome assembly and regulation. *Nature* **559**, 419-422 (2018).

3. Fianu I*, et al.* Structural basis of Integrator-dependent RNA polymerase II termination. *Nature* **629**, 219-227 (2024).
