# Supplementary material for: Structure of a transcribing Pol II-DSIF-SPT6-U1 snRNP complex
Source: Nat Commun. 2025 Jul 1;16:5823. doi: 10.1038/s41467-025-60979-9 (PMC12216098; doi:10.1038/s41467-025-60979-9)
Supplement: Supplementary file 2 — Description of Additional Supplementary Files [file 41467_2025_60979_MOESM2_ESM.pdf]

Description of Additional Supplementary Files

File name: Supplementary Movie 1
Description: The transcription elongation complex recruits U1 snRNP through multiple interaction sites.
